# Neuroprotection: Pro-survival and Anti-neurotoxic Mechanisms as Therapeutic Strategies in Neurodegeneration

**DOI:** 10.3389/fncel.2019.00231

**Published:** 2019-06-06

**Authors:** Horacio Uri Saragovi, Alba Galan, Leonard A. Levin

**Affiliations:** ^1^Lady Davis Institute, Montreal, QC, Canada; ^2^Jewish General Hospital, Montreal, QC, Canada; ^3^Department of Ophthalmology and Visual Sciences, McGill University, Montreal, QC, Canada; ^4^McGill University Health Centre, Montreal, QC, Canada; ^5^Montreal Neurological Institute, Mcgill University, Montreal, QC, Canada

**Keywords:** small molecule, mimetic, antibody, growth factor, neurotrophin, receptor, neurodegeneration, therapy

## Abstract

Neurotrophins (NTs) are a subset of the neurotrophic factor family. These growth factors were originally named based on the nerve growth functional assays used to identify them. NTs act as paracrine or autocrine factors for cells expressing NT receptors. The receptors and their function have been studied primarily in cells of the nervous system, but are also present in the cardiovascular, endocrine, and immune systems, as well as in many neoplastic cells. The signals activated by NTs can be varied, depending on cellular stage and context, healthy or disease states, and depending on whether the specific NTs and their receptors are expressed in the relevant cells. In the healthy central and peripheral adult nervous systems, NTs drive neuronal survival, phenotype, synaptic maintenance, and function. Deficiencies of the NT/NT receptor axis are causally associated with disease onset or disease progression. Paradoxically, NTs can also drive synaptic loss and neuronal death. In the embryonic stage this activity is essential for proper developmental pruning of the nervous system, but in the adult it can be associated with neurodegenerative disease. Given their key role in neuronal survival and death, NTs and NT receptors have long been considered therapeutic targets to achieve neuroprotection. The first neuroprotective approaches consisted of enhancing neuronal survival signals using NTs. Later strategies selectively targeted receptors to induce survival signals specifically, while avoiding activation of death signals. Recently, the concept of selectively targeting receptors to reduce neuronal death signals has emerged. Here, we review the rationale of each neuroprotective strategy with respect to the complex cell biology and pharmacology of each target receptor.

## Introduction

Neurotrophins (NTs) are a family of growth factors that include nerve growth factor (NGF), brain-derived neurotrophic factor (BDNF), neurotrophin-3 (NT-3), and neurotrophin 4/5 (NT-4/5). Neurotrophin mRNAs are translated into larger precursors named pro-NTs, which are then processed into mature NTs by enzymatic cleavage. The pro-NTs and the mature NTs activate different and sometimes opposing signals and physiological pathways, acting through different receptors (Teng, and Hempstead, 2004).

Mature NTs preferentially activate Trk receptors, which are generally associated with survival signals, whereas pro-NTs activate p75 receptors, which are generally associated with death signals (Chao et al., 2006). Neurodegenerative diseases often exhibit imbalances either in NT (e.g., poor processing of pro-NTs to the mature state or poor transport of NTs to the site, where they are needed) or imbalances in NT receptors (e.g., decreases in pro-survival Trks and increases in pro-death p75). This summary is a simplification, and there are additional issues, such as positive and negative functional cross-regulation of Trks and p75, when these receptors are co-expressed. In certain cellular contexts the receptors may also have altered functions, for example in neoplasia.

Clinical trials using NTs as drugs to promote neuroprotection have consistently failed, in large part due to poor receptor selectivity (e.g., binding to Trks and p75), their pleiotropic pro-survival and pro-death activities, short half-lives, poor pharmacokinetics and bioavailability (Yuen and Mobley, 1996; Thoenen and Sendtner, 2002), or because pro-death p75 receptors may be up-regulated in disease (Rudzinski et al., 2004; Ibanez and Simi, 2012), thus negating the full benefit of Trk activation (Josephy-Hernandez et al., 2017). In addition, in some cases Trk receptors can also promote neuronal death (Tauszig-Delamasure et al., 2007), resulting in a confusing pattern of *in vivo* physiology and hard-to-predict pharmacology.

Many excellent reviews of the NT field discuss the biology and physiology of each factor and receptor (Ibanez and Simi, 2012; Bothwell, 2016), and postulate reasons to explain clinical failures (Yuen and Mobley, 1996; Thoenen and Sendtner, 2002). Other comprehensive reviews describe compounds reported to activate/inactivate Trks and p75 receptors, some of which were used as proof-of-concept therapeutics [reviewed in (Longo and Massa, 2004; Josephy-Hernandez et al., 2017)].

Here, we present a reassessment of neuroprotection strategies and their challenges, focusing on the paradoxes of receptor pharmacology and signals. We distinguish strategies that promote survival and strategies that reduce neurotoxicity, as separate but complementary approaches. Each strategy faces challenges which must be addressed for successful translation into clinically effective neuroprotective therapies.

## Neurotrophin Receptors and Roles in Disease

**FIGURE 1:**
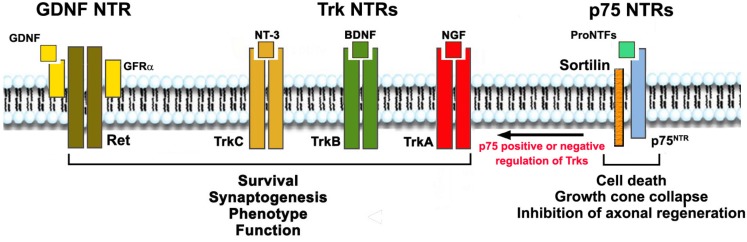
Neurotrophic Factors, their receptors (NTRs) and functions. NTs activate signaling receptors TrkA, TrkB, TrkC, and p75^NTR^. Each NT receptor (NTR) can act alone, and all Trks can also cooperate positively or negatively with p75^NTR^. GDNF acts through RET but binds the glycosyl-phosphatidylinositol-anchored co-receptor (GFRα1–4) subunits. Mature NTs (NGF, BDNF, NT-3) bind, respectively, TrkA, TrkB and TrkC with some selectivity. All NTs and the precursor pro-neurotrophin proteins bind to p75^NTR^. Growth factor activation of Trks or RET promote survival, growth, differentiation and synaptogenesis. Trk receptors may be present in truncated forms that are not protective (not shown). Growth factor activation of p75^NTR^/sortilin complex in neurons leads to apoptosis, growth cone collapse, and inhibition of axonal regeneration; and in glia, activation of p75^NTR^ leads to production of pro-inflammatory and neurotoxic factors. p75^NTR^ can also regulate the action of Trk receptors positively or negatively, depending on its binding partners and other factors.

Neurotrophins (NTs) act through two distinct receptor families: three receptor tyrosine kinases named TrkA TrkB and TrkC, and a receptor named p75. NGF binds TrkA, BDNF and NT-4 bind TrkB, and NT-3 prefers TrkC but also can bind TrkA and TrkB (Figure 1).

**Table 1:** Experimental (and in some cases clinical) validation of the indicated receptor targets as “proof-of-concept” in neurodegenerative diseases affecting the CNS.

	TrkC	TrkC.T1	p75	TrkA	TrkB	TrkB.T1	RET
**Brain / Spinal Cord**							
Alzheimer’s/memory			Antag.	Agonist	Agonist	Prevent splicing^∗^	
Parkinson’s			?		Agonist		Agonist
ALS	Agonist	Antag. or prevent splicing^∗^	Antag.		Agonist	Prevent splicing^∗^	Agonist
Huntington’s			Antag.		Agonist		Agonist
**Cochlea**							
Hearing loss	Agonist	Antag. or prevent splicing^∗^	?		Agonist		Agonist
**Retina**							
Retinitis pigmentosa		Antag.	Antag.				Agonist
Glaucoma		Antag. or prevent splicing^∗^	Antag.	Agonist	Agonist		Agonist
Diabetic retinopathy			Antag.				
Optic nerve injury			Antag.	Agonist	Agonist		Agonist
Retinal angiogenesis		Antag.	Antag.				

*Antag = antagonist. ALS = amyotrophic lateral sclerosis. ^∗^Prevention of TrkB or TrkC mRNA splicing to their truncated isoforms was achieved by an engineered mutation of the splicing site in transgenic mice. ? = unclear or contradictory data.*

Ligands binding to Trks drive the activation of the receptor tyrosine kinase enzymatic activity and the tyrosine phosphorylation of intracellular proteins (e.g., PLCγ, PI3K, Ras and Raf/MEK/Erk1) to initiate signaling pathways (Saragovi and Gehring, 2000; Miller and Kaplan, 2001). These intracellular signaling pathways are usually associated with neuronal survival, maintenance and function in the peripheral and central nervous systems (CNS), and the survival of stressed neurons (Huang and Reichardt, 2003). Therefore, activation of Trks or their signaling cascades has been sought as a mechanism to thwart neuronal degeneration (Saragovi and Gehring, 2000; Saragovi et al., 2009).

All NTs, including the pro-NTs, also bind to p75, a member of the Tumor Necrosis Factor-α (TNFα) receptor superfamily. NTs binding to p75 lead to the activation of complex cascades that are stage- or tissue-specific. Generally, p75 is believed to signal neuronal atrophy, synaptic loss, loss of function, and cell death (Zaccaro et al., 2001; Hempstead, 2002, 2006; Saragovi and Zaccaro, 2002; Ivanisevic et al., 2003; Chao et al., 2006; Saragovi et al., 2009), and is commonly upregulated in neurodegenerative diseases. In pathological states (Table 1), pro-NTs binding to p75 promotes the production of inflammatory cytokines TNFα and α2 Macroglobulin (α2M) by glial cells, and increase levels of pro-NGF itself, thus perpetuating the deleterious activation of p75. All of these factors are neurotoxic at increased levels, and result in a vicious cycle of neurotoxic events (Barcelona and Saragovi, 2015).

Adding another layer of complexity to this signaling axis, the biological function of p75 depends on several factors. The p75 receptor cooperates with other proteins, such as the sortilin family of receptors, which are integral to p75 signaling (Vaegter et al., 2011; Skeldal et al., 2012). Additionally, co-expression of Trks can influence p75 biology, as can the type of cell and its stage of maturation/differentiation. As mentioned above, the p75 receptor also has multiple ligands (all NTs as well as all pro-NTs) (Dechant and Barde, 2002; Nykjaer et al., 2005; Al-Shawi et al., 2007), and co-expression of p75 and Trks regulates ligand binding and affinity (Hempstead and Chao, 1997), as well as functional signaling (Zaccaro et al., 2001; Saragovi and Zaccaro, 2002) in a positive or negative manner (Figure 1). Due to the complexity of the signaling pathways, the consequences of pharmacological modulation of p75 *in vivo* are difficult to predict.

Yet another level of complexity in Trk receptor signaling emerges from the presence of Trk receptor isoforms. Trks can be expressed as full-length Trk tyrosine kinase receptors (Trk-FL) or as truncated isoforms without a kinase domain. The truncated isoforms are generated by alternative mRNA splicing, gaining a new and distinct intracellular sequence while lacking intrinsic tyrosine kinase activity (Brahimi et al., 2016). Both truncated TrkB.T1 and TrkC.T1 are able to mediate signals, either by inhibiting full-length tyrosine kinase as a dominant-negative mechanism (Valenzuela et al., 1993; Palko et al., 1999; Das et al., 2000) or by being activated in a ligand-dependent manner (Baxter et al., 1997; Esteban et al., 2006). Pathological upregulation of TrkC.T1 induces neuronal cell death through activation of Rac1 GTPase and pERK signaling pathways, with subsequent increase of TNFα production to toxic levels (Esteban et al., 2006; Galán et al., 2017b). Expression of TrkB.T1 and TrkC.T1 receptors increases in many neurodegenerative diseases (Table 1; Bai et al., 2010b; Yanpallewar et al., 2012; Galán et al., 2017b). Given that truncated isoforms are increased in disease and can be activated by NTs to cause toxicity, it would seem counterintuitive to use NTs as therapeutic agents in these conditions.

## Neurotrophic Factors in Clinical Development

Neurotrophins (NTs) are part of a larger family of neurotrophic factors (NTFs). Among the NTFs, glial-derived neurotrophic factor (GDNF), ciliary-derived neurotrophic factor (CNTF), and insulin-like growth factor-1 (IGF-1) are key determinants of neuronal health (Sakowski et al., 2009; Pasquin et al., 2015) and were tested for clinical efficacy (Yuen and Mobley, 1996; Thoenen and Sendtner, 2002).

Glial-derived neurotrophic factor (GDNF) signals by binding to a co-receptor known as GFRα1, leading to the activation of the tyrosine kinase Ret. Similarly to its counterparts in the Trk family, Ret induces survival through the PI3K/Akt, Ras/Erk, Src and PLCγ signaling pathways (Ibanez, 2013). The absolute requirement of GFRα co-receptors for GDNF activation of Ret limits the number of neurons targeted by GDNF.

## The Evolution of the Concept of NT-Based Neuroprotection

The translational potential of NTs as drugs and NT receptors as targets for neuroprotection has long been recognized, and several clinical trials were carried out in the 1990’s, none resulting in regulatory approval, the main problems attributed to lack of specific targeting, not achieving an effective dose, and failure to avoid side effects (Thoenen and Sendtner, 2002). The failure of these trials to achieve their endpoints led to a re-evaluation of the validity and druggability of these targets in disease, and particularly the scrutiny of the physiological basis for their pharmacology.

**FIGURE 2:**
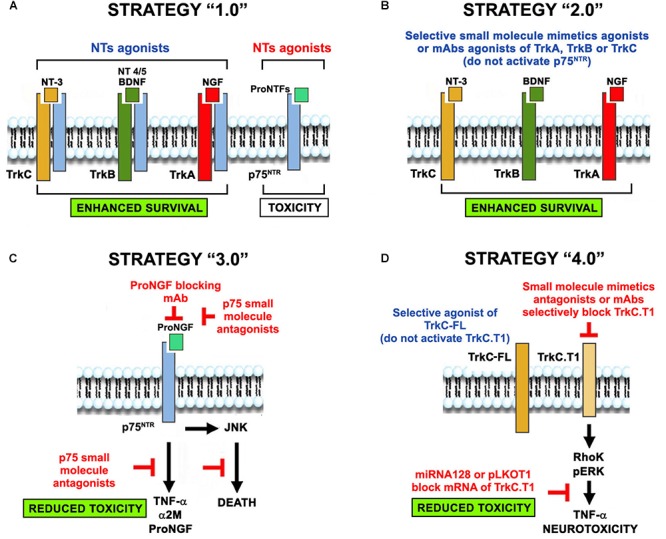
The evolution of the concept of NT-based neuroprotection. Four neuroprotective strategies evolved from the use of NTs-based therapies in neurodegenerative diseases. **(A)** Neuroprotection strategy 1.0 was the original use of NTs regardless of receptor selectivity, causing unintended signaling through p75^NTR^ or truncated Trk isoforms that are up-regulated in neurodegenerative pathologies. **(B)** Neuroprotection strategy 2.0 overcomes some of the drawbacks by using selective agonists of Trk receptors (small molecules, mAbs, and mutant NTs that do not bind p75), circumventing p75^NTR^ activation. **(C)** Neuroprotection strategy 3.0 addresses p75^NTR^ neurotoxicity using selective blocking antibodies and small molecules antagonists. **(D)** Neuroprotection strategy 4.0 addresses the toxic function of truncated Trk isoforms by using selective Trk full-length agonists (that do not activate the truncated forms) or selective antagonists of truncated isoforms (small molecules, mAbs, miRNAs and shRNA vectors). Co-expression of p75 and TrkC.T1 in glia may exacerbate neurotoxicity. We predict that combinations of these strategies (e.g., strategy 2 + strategy 3) may be synergistic because of their complementary mechanisms of action.

Below we present evolving concepts of NT receptor biology and receptor physiology relevant to their roles in disease. We discuss 4 generations of neuroprotection strategies. Neuroprotection strategy 1.0 refers to the use of NTs or NT-inducing agents regardless of receptor selectivity, pharmacokinetics, or receptor expression patterns. Neuroprotection strategy 2.0 refers to the selective activation of Trk receptors without p75 binding and activation, particularly in diseases, where p75 is upregulated. Neuroprotection strategy 3.0 refers to inhibition of p75-mediated signals, which are pro-inflammatory and neurotoxic. Neuroprotection strategy 4.0 refers to improved Trk-activation in a selective manner, not only circumventing p75 activation but also the activation of truncated Trk isoforms that mediate neurotoxicity (Figure 2). Conceivably, these 4 strategies may be combined to achieve synergy, given that they have different mechanisms of action.

## Neuroprotection Strategy 1.0: Activating NT Receptors

Neurotrophins (NTs) normally drive neuronal survival, maintenance of phenotype and synapses, and function. Ligand-dependent activation of the Trk receptors is associated with those survival signals (Sendtner et al., 1996; Bechade et al., 2002; Wan et al., 2014; Brahimi et al., 2016). As mentioned earlier, deficits in the activation of Trk receptor tyrosine kinases (for instance, impaired cellular transport, decreased receptor expression, or agonist deficiency) are linked to early stages of neurodegeneration, and precede neuronal death and symptoms. These data supported the rationale that Trk-agonism may be therapeutic, and NTs were evaluated in multiple experimental models with the expectation that they would solely act as Trk agonists.

Nerve growth factor (NGF) was studied therapeutically to activate TrkA in models of Alzheimer disease (AD) (Schindowski et al., 2008; Cuello et al., 2010), and ageing (Mufson et al., 2000; Bruno et al., 2004; Saragovi, 2005), or Down syndrome (Sendera et al., 2000; Dorsey et al., 2006), models with cholinergic deficits and memory impairment. BDNF and GDNF were studied therapeutically in models of Parkinson disease (PD) (Rangasamy et al., 2010) and Huntington disease (HD) (Alberch et al., 2004), with loss of neurons that express TrkB and RET. NT-3 was studied therapeutically in models of amyotrophic lateral sclerosis (ALS) with loss of spinal cord motor neurons that express TrkC (Ekestern, 2004). Ophthalmic neurodegenerative diseases such as glaucoma (Rudzinski et al., 2004; Nafissi and Foldvari, 2016), retinitis pigmentosa (RP) (Thanos and Emerich, 2005), and diabetic retinopathy (DR) (Bikbova et al., 2014) were studied using NTs as therapeutic agents. In most of these diseases, growth factors other than NTs were explored, including GDNF, CNTF, and IGF-1 (Kaspar et al., 2003; Dupraz et al., 2013; Bassil et al., 2014; Ma et al., 2015; Agostinone et al., 2018) [reviewed in (Gould and Oppenheim, 2011)].

Clinical trials using exogenous delivery of these factors or cells secreting these factors, designed to agonize Trk or RET or IGF-1 receptors, have been consistently unsuccessful. Reasons include the poor pharmacokinetics and pharmacodynamics of NTs, short half-lives, undesirable high potency and pleiotropic effects, inability to penetrate tissue barriers, and difficulty in delivery of these large proteins across the BBB, requiring increasingly sophisticated and risky methods of administration (Weissmiller and Wu, 2012; Rafii et al., 2018), with consequent limitations in reaching the relatively high doses required for efficacy in experimental studies.

Notably, the reasons thought to be responsible for failure are not exclusive to NTs: they generally affect most protein-based therapies, and the issues are resolvable. But there are problems specific to NTs. These include poor receptor selectivity and unpredictable *in vivo* pharmacology, particularly off-target effects on unintended activation of p75 or truncated Trk receptors (Saragovi and Gehring, 2000; Josephy-Hernandez et al., 2017). The expression and activity of these receptors are increased in neurodegenerative states, and given that they are neurotoxic, they invalidate the benefit of Trk activation, decrease the therapeutic effect, and create a poor risk/benefit ratio (Figure 2A; Peleshok and Saragovi, 2006; Josephy-Hernandez et al., 2017).

## Neuroprotection Strategy 2.0: Targeting Full Length Trk Receptors and Avoiding p75 Activation

Based on the many failures of neuroprotection strategy 1.0, we hypothesized that selective Trk-activating agents which circumvent p75 binding and activation would be neuroprotective. To test this idea, we produced a wide range of TrkA-, TrkB-, and TrkC-selective agonists and tested them in different neurodegenerative pathologies (Figure 2B). GDNF agonists are also briefly summarized.

### TrkA-, TrkB-, or TrkC-Selective Agonists

We generated small molecule mimetics of NTs (LeSauteur et al., 1995, 1996a; Debeir et al., 1999; Maliartchouk et al., 2000a,b; Pattarawarapan et al., 2002; Zaccaro et al., 2005; Chen et al., 2009), agonistic mAbs (LeSauteur et al., 1996b, Saragovi et al., 1998; Bai et al., 2010c; Guillemard et al., 2010), small molecule mimetics of the mAbs (Brahimi et al., 2009, 2010, 2014; Chen et al., 2009), and mutant NTs (Saragovi et al., 1998; Ivanisevic et al., 2007; Bai et al., 2010a; Aboulkassim et al., 2011). We then tested them in animal models of neurodegenerative disease, demonstrating effectiveness *in vivo*.

For instance, a TrkA-selective NGF mutant that does not activate p75 (Bai et al., 2010a) and the selective small molecule TrkA agonist D3 (Shi et al., 2007) rescued retinal ganglion cells (RGCs) in glaucoma and optic nerve axotomy models. A selective agonistic mAb that binds TrkB delayed RGC death and preserved the structure of retinal layers from degenerating in optic nerve axotomy and glaucoma (Bai et al., 2010c). A mutant NT-3 selective for TrkC, and an agonistic mAb that activates TrkC (Guillemard et al., 2010) selectively protected motor neurons in an ALS model (Enomoto et al., 2013; Brahimi et al., 2016). None of these ligands bind or activate p75. Many of these agents were very effective *in vivo* in disease states and paradigms, where the native NTs were ineffective unless p75 was concomitantly silenced or neutralized (Bruno et al., 2004; Shi et al., 2007; Lebrun-Julien et al., 2009b, 2010).

Later, other groups validated the concept by generating TrkB small molecule agonists and TrkB and TrkC agonistic mAbs that were protective in the MPTP neurotoxicity mouse model of PD (Berezov et al., 2002; Jang et al., 2010; Devi and Ohno, 2012; Bollen et al., 2013; Coles et al., 2014; Chitranshi et al., 2015; Nie et al., 2015), HD (Jiang et al., 2013; Simmons et al., 2013), and ALS. These agents were effective in experimental paradigms, where the wild type NTs were ineffective, whether endogenously produced or added as therapeutic agents. Yet the wild type NTs became effective when p75 expression was concomitantly silenced or inhibited [reviewed in Josephy-Hernandez et al. (2017)].

With respect to translation to clinical use, one of our TrkA-selective small molecule agonists is currently in Phase 3 clinical trials for an ophthalmic indication, a TrkC-selective mAb agonist is in pre-clinical studies for ALS, and Trk-selective agonistic mAbs and NT mutants are under investigation for neurosensory hearing loss. Other translational efforts with Trk-selective agonists are in progress in PD, ALS, AD, and HD.

### GDNF/Ret Agonists

The GDNF/Ret/GFRα1 axis plays an important neuroprotective role in the retina and other anatomical sites. GDNF causes upregulation of the glutamate aspartate transporter in glial cells, and therefore counteracts the excitotoxic environment in the degenerating retina (Delyfer et al., 2005). GDNF also stimulates the secretion of osteopontin and basic fibroblast growth factor (bFGF), which have been shown to prolong rod survival (Hauck et al., 2006; Del Rio et al., 2011). Following the initial evaluation of GDNF therapies (Strategy 1.0), new efforts have led to the generation of small-molecule agonists with GDNF-like activity or Ret modulatory activity (Sidorova et al., 2017).

Norgestrel is a small molecule related to progesterone that has been demonstrated to be neuroprotective in the rd10 model of RP (Doonan et al., 2011), possibly through the upregulation of bFGF. XIB4035t is a small molecule with effects on GFRα1/Ret signaling. Originally, it was inappropriately characterized as a GFRα1 agonist (Tokugawa et al., 2003), but is nowadays considered to be a GFRα1 modulator, able to potentiate signaling in the presence of GDNF (Hedstrom et al., 2014; Sidorova et al., 2017; Ivanova et al., 2018).

## Neuroprotection Strategy 3.0: Inhibiting p75 Receptors

In the adult, the p75 receptor is expressed at low levels in healthy states (Tomellini et al., 2014), but is upregulated in disease. This injury-induced expression recapitulates the role of p75 in development (Ibanez and Simi, 2012), where p75 is expressed at high levels and modulates synaptic pruning and the death of unwanted neurons (Lebrun-Julien et al., 2009b; Bai et al., 2010b). The upregulated p75 receptor is generally associated with neuronal death and is activated primarily by proNGF (Figure 2C; Teng et al., 2005).

In neurons, activation of p75 by proNGF triggers apoptotic death, decreases synaptic function (Jansen et al., 2007), and reduces the neuroprotective effect associated with agonists of full-length Trk receptors (Zaccaro et al., 2001; Saragovi and Zaccaro, 2002; Ivanisevic et al., 2003; Saragovi et al., 2009).

In the vasculature, p75 activation causes pericyte dysfunction (Siao et al., 2013; Barcelona et al., 2016) and breakdown of blood–tissue barriers, causing vascular permeability and edema, and leading to vascular endothelial cell death and hypoxia (Shanab et al., 2015). Consequent vaso-obliteration and hypoxia induce VEGF, angiogenic remodeling, and pathological neovascularization (Siao et al., 2013; Barcelona et al., 2016). Thus, p75 on pericytes is relevant to deficits after cardiac hypoxia associated with cardiac injury, retinal neovascularization in DR, and choroidal neovascularization in the wet form of age-related macular degeneration.

In glia, p75 enhances production of the inflammatory mediators TNFα (Srinivasan et al., 2004; Nakazawa et al., 2006; Lebrun-Julien et al., 2009a, Lebrun-Julien et al., 2010; Bai et al., 2010b; Barcelona et al., 2016; Galan et al., 2017a; Platon-Corchado et al., 2017), proNGF (Srinivasan et al., 2004; Lim et al., 2008; Lebrun-Julien et al., 2009b, 2010; Xu et al., 2009; Barcelona et al., 2016), and α2M (Shi et al., 2008; Bai et al., 2010a,b; Barcelona et al., 2016; Galan et al., 2017a; Platon-Corchado et al., 2017). Each of these factors is neurotoxic, and they cooperate to synergistically worsen pathology (Mysona et al., 2013; Barcelona and Saragovi, 2015). Mechanistically, α2M extends the half-lives of TNFα and proNGF (Barcelona and Saragovi, 2015), and p75-driven production of proNGF generates an autocrine loop, resulting in its persistent activation.

Notably, TNFα, proNGF, and α2M are each validated therapeutic targets. Inhibition of TNFα (Nakazawa et al., 2006; Roh et al., 2012), proNGF (Barcelona et al., 2013; Barcelona and Saragovi, 2015), or α2M (Shi et al., 2008; Bai et al., 2010a,b) as monotherapy affords moderate efficacy in retinal neurodegeneration, and other pathologies. Thus, p75 overexpression in disease creates an unfavorable environment by driving at least three neurotoxic proteins, which then feeds back in an autocrine-mediated vicious cycle. Expression and activity of p75 in disease disrupts neuro-glia-vascular homeostasis, with progressive pathology, whereby inflammation causes neuronal death, and neuronal death causes more inflammation and vascular pathology.

These observations in animal models are relevant to human neurodegenerative and vascular diseases because increased levels of p75, TNFα, proNGF, and α2M, alone or in combination, have been documented in ALS, cardiac hypoxia, RP, DR, and others. Inhibition of p75 should also prevent direct neuronal death, vascular deficits, acute inflammation, and chronic production of TNFα, proNGF, and α2M.

We developed a family of drug-like agents that selectively inhibit p75 activity (Bai et al., 2010a; Josephy-Hernandez et al., 2017). These p75 antagonists were therapeutic in models of glaucoma and optic nerve injury (Bai et al., 2010a), RP (Galán et al., 2017b; Platon-Corchado et al., 2017), and neovascularization in DR (Barcelona et al., 2016; Galan et al., 2017a), even when applied as monotherapy after disease onset. Other small p75 ligands reduced tau-related pathology in AD (Massa et al., 2002; Nguyen et al., 2014) and p75-induced motor neuron cell death in ALS (Pehar et al., 2006).

## Neuroprotection Strategy 4.0: Inhibiting Truncated Trk Receptors

As described above, the full length Trk receptors have a tyrosine kinase intracellular domain that phosphorylates and activates survival signals such as Akt or PLC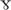 pathways in a ligand-dependent manner. The “full length” (FL) receptor activity is critical to the survival and function of neurons, where they are expressed. For instance, TrkC-FL is necessary for the physiology of motor neurons and cochlear neurons (Sendtner et al., 1996; Bechade et al., 2002; Mellado Lagarde et al., 2014; Wan et al., 2014; Wan and Corfas, 2015; Brahimi et al., 2016; Suzuki et al., 2016), whereas TrkB-FL is necessary for brain dopaminergic neurons (Nie et al., 2015).

However, there are truncated Trk receptors which arise from alternative mRNA splicing of Trk-FL mRNAs. The resulting truncated isoforms lack the kinase domain, and signal differently. The most common truncated TrkC and TrkB isoforms in humans and rodents are TrkC.T1 (Tessarollo, 1998; Palko et al., 1999) and TrkB.T1, respectively, (Figure 2D; Yanpallewar et al., 2012).

### Truncated TrkC Isoform (TrkC.T1)

TrkC.T1 arises from alternative mRNA splicing of TrkC-FL mRNA, resulting in deletion of the kinase domain, and gain of a new intracellular domain with a unique sequence from a spliced-in exon. For this reason, the TrkC-FL and TrkC.T1 isoforms only differ at the intracellular domain primary sequences, but retain the same extracellular primary sequence. Both TrkC-FL and TrkC.T1 bind NT-3 equally well and both signal in an NT-3–dependent manner (Esteban et al., 2006).

TrkC.T1 acts by activation of Rho kinase-Erk pathways (Esteban et al., 2006; Galán et al., 2017b), and via tamalin (Esteban et al., 2006). One physiological consequence of TrkC.T1 activity is an NT-3–dependent increase in TNFα, which is neurotoxic (Bai et al., 2010b; Brahimi et al., 2016; Galán et al., 2017b). Hence there must be a balance of TrkC-FL and TrkC.T1 signals in health and disease. In healthy adult tissue TrkC.T1 is low or undetectable, but it is significantly upregulated shortly after injury, but before degeneration and detectable symptoms, in neurodegenerative diseases such as glaucoma, RP, and ALS (Bai et al., 2010b; Brahimi et al., 2016; Galán et al., 2017b), as well as in noise-induced hearing loss (Saragovi, unpublished).

TrkC.T1 mRNA is produced constitutively and is immediately degraded by miRNA128. miRNA128 is reduced and TrkC.T1 mRNA is increased in neurodegenerative diseases (Brahimi et al., 2016). Hence, at the onset of neurodegenerative diseases TrkC.T1 protein is upregulated, but without a decrease in TrkC-FL until very late in disease, when neurons die (Bai et al., 2010b; Brahimi et al., 2016; Galán et al., 2017b).

TrkC-FL is present mainly in neurons, whereas in disease states TrkC.T1 is mainly in glia and astrocytes. Hence the TrkC.FL/TrkC.T1 ratio is reduced in diseased tissues with a difference in cellular distribution that is relevant to the mechanisms of action. In glia, TrkC.T1 activity promotes TNFα production in a pErk–dependent manner, and *in vivo* all TrkC.T1 mRNA co-localizes in cells expressing TNFα mRNA (Brahimi et al., 2016; Galan et al., 2017a; Galán et al., 2017b). The TrkC.T1–dependent increase in TNFα is neurotoxic and neurodegenerative, and relevant to the etiology of glaucoma and RP (Bai et al., 2010b; Galán et al., 2017b).

NT-3 binds TrkC-FL and TrkC.T1, and therefore paradoxically activates both neuroprotective and neurodegenerative signals. In this context, we postulated that NT-3 would be therapeutically useful if administered before disease onset (e.g., before TrkC.T1 upregulation), whereas neurotoxicity would predominate when NT-3 was applied after disease onset (e.g., after TrkC.T1 upregulation) (Corse et al., 1999; Park et al., 2009; Wyatt et al., 2011; Suzuki et al., 2016). Indeed, this paradox has been reported in motor neuron degeneration (such as SOD1 mutant rodent models of ALS) (Brahimi et al., 2016), in the death of retinal ganglion cell neurons in a model of RP (Galán et al., 2017b), and may be germane to the death of spiral ganglion neurons in a noise-induced hearing loss model (NIHL).

To directly test the hypothesis that the therapeutic failure of NT-3 is due in part to the unintended activation of TrkC.T1, we developed selective agonists of TrkC-FL. These agents activate TrkC-FL but do not bind or activate TrkC.T1, and therefore do not stimulate TNFα production (Guillemard et al., 2010; Brahimi et al., 2016). In an animal model of ALS these selective TrkC-FL agonists protect motor neuron health and significantly prolong life-span, even when injected after disease onset (Brahimi et al., 2016).

Additionally, we validated this concept by showing that in disease models of neurodegeneration (glaucoma causing RGC neuronal death (Bai et al., 2010b) and a genetically driven model of RP causing photoreceptor neuronal death (Galán et al., 2017b), reduced TrkC.T1 expression had significantly reduced disease progression. reduced levels of TNFα, lower activation of pErk in glia, and reduced neuronal death and neurodegeneration.

With respect to pharmacological inhibition, we have also developed highly selective inhibitors of TrkC.T1 expression (miRNA128 and shRNA vectors) which silence or inhibit TrkC.T1 and prevent induction of TNFα by NT-3 *in vitro* (Brahimi et al., 2016) and *ex vivo* in organotypic cultures (Galán et al., 2017b). Published small molecule inhibitors of TrkC (Brahimi et al., 2010, Brahimi et al., 2014, 2016; Liu et al., 2010) are non-selective between TrkC-FL and TrkC.T1, but significantly decrease TNFα levels and neuronal cell death in a mouse model of glaucoma (Bai et al., 2010b). The non-selective TrkC antagonists used in glaucoma were useful because virtually all the TrkC in the glaucomatous retina is TrkC.T1. However, selective TrkC.T1 inhibitors would be preferable, and we have developed and evaluated such agents (Brahimi et al., 2009, Brahimi et al., 2016).

### Truncated TrkB Isoform (TrkB.T1)

TrkB.T1 (T1) is the main TrkB isoform in the mature brain (Dorsey et al., 2006) and its function was studied *in vivo* (Ferrer et al., 1999). Similar to TrkC.T1, TrkB.T1 inhibits TrkB.FL signaling acting as a dominant-negative receptor, thereby decreasing the effects of BDNF on neuronal survival, differentiation, and plasticity. TrkB.T1 also has BDNF-independent functions and regulates Rho GTPase activity (Ohira et al., 2006) and may stimulate PLCγ and MAPK signaling (Ohira et al., 2007).

TrkB.T1 is upregulated in neurodegenerative diseases such as AD (Ferrer et al., 1999) and ALS (Yanpallewar et al., 2012). Genetic deletion of TrkB.T1 in the SOD mouse model of ALS significantly delayed the onset of motor neuron degeneration (Yanpallewar et al., 2012) and restored cognitive abnormalities (Quarta et al., 2018).

In summary, TrkC.T1 (along with p75, TNFα, proNGF, and α2M) and TrkB.T1 are upregulated in animal and in human diseases (Sanchez et al., 2007; Bai et al., 2011; Roh et al., 2012; Yanpallewar et al., 2012; Siao et al., 2013; Shepheard et al., 2014) and are relevant to pathophysiology, making them excellent therapeutic targets.

## Conclusion

It has been 25 years since the first clinical trials of NTs in CNS neurodegenerative disorders. These and subsequent studies failed, due not only to poor pharmacokinetics, short half-lives and/or poor bioavailability of the drugs, but also due to the complex biology of NTs and their receptors. Our understanding of the finely tuned physiology of NT receptors is critical to developing strategies for selectively restoring the balance between neuroprotective and neurotoxic signals. Over the years, we have learned that failures of NTs (neuroprotection strategy 1.0) were related to their pleiotropic effects, poor selectivity and off-target effects on unintended p75 or truncated Trk isoforms. More specific and successful strategies were developed using specific Trk-activating agents that circumvented p75 (neuroprotection strategy 2.0) and therefore potentiated neuroprotection, or by specifically inhibiting p75 receptors (neuroprotection strategy 3.0) or truncated Trk isoforms (neuroprotection strategy 4.0), both signaling neurotoxicity.

NT-based neuroprotection is still an evolving concept, and we can expect the development of even more focused strategies in coming years, which should deal with the complex nature of NT receptor physiology. We envision a future neuroprotection strategy 5.0 based on combining strategies, for example a synergistic neuroprotective and anti-neurotoxic combination, which might finally provide successful translation for treatment of chronic neurodegenerative diseases.

## Author Contributions

All authors listed have made a substantial, direct and intellectual contribution to the work, and approved it for publication.

## Conflict of Interest Statement

The authors declare that the research was conducted in the absence of any commercial or financial relationships that could be construed as a potential conflict of interest.
